# Patterns of Use of a Price Transparency Tool for Childbirth Among Pregnant Individuals With Commercial Insurance

**DOI:** 10.1001/jamanetworkopen.2021.21410

**Published:** 2021-08-18

**Authors:** Rebecca A. Gourevitch, Alyna T. Chien, Elizabeth A. Bambury, Neel T. Shah, Christine Riedl, Meredith B. Rosenthal, Anna D. Sinaiko

**Affiliations:** 1 Department of Health Care Policy, Harvard Medical School, Boston, Massachusetts; 2 Division of General Pediatrics, Department of Medicine, Boston Children’s Hospital, Boston, Massachusetts; 3 Department of Pediatrics, Harvard Medical School, Boston, Massachusetts; 4 Department of Health Policy and Management, Harvard T.H. Chan School of Public Health, Boston, Massachusetts; 5 Ariadne Labs, Boston, Massachusetts; 6 Department of Obstetrics and Gynecology, Beth Israel Deaconess Medical Center, Boston, Massachusetts; 7 Retired

## Abstract

**Importance:**

When introduced a decade ago, patient-facing price transparency tools had low use rates and were largely not associated with changes in spending. Little is known about how such tools are used by pregnant individuals in anticipation of childbirth, a shoppable service with increasing out-of-pocket spending.

**Objective:**

To measure changes over time in the patterns and characteristics of use of a price transparency tool by pregnant individuals, and to identify the association between price transparency tool use, coinsurance, and childbirth spending.

**Design, Setting, and Participants:**

This descriptive cross-sectional study of 2 cohorts used data from a US commercial health insurance company that launched a web-based price transparency tool in 2010. Data on all price transparency tool queries for 2 periods (January 1, 2011, to December 31, 2012, and January 1, 2015, to December 31, 2016) were obtained. The sample included enrollees aged 19 to 45 years who had a delivery episode during 2 periods (November 1, 2011, to December 31, 2012, or November 1, 2015, to December 31, 2016) and were continuously enrolled for the 10 months prior to delivery (N = 253 606).

**Exposures:**

Access to a web-based price transparency tool that provided individualized out-of-pocket price estimates for vaginal and cesarean deliveries.

**Main Outcomes and Measures:**

The primary outcomes were searches on the price transparency tool by delivery mode (vaginal or cesarean), timing (first, second, or third trimester), and individual characteristics (age at childbirth, rurality, pregnancy risk status, coinsurance exposure, area educational attainment, and area median household income). Another outcome was the association of out-of-pocket childbirth spending with price transparency tool use.

**Results:**

The sample included 253 606 pregnant individuals, of whom 131 224 (51.7%) were in the 2011 to 2012 cohort and 122 382 (48.3%) were in the 2015 to 2016 cohort. In the 2015 to 2016 cohort, the mean (SD) age was 31 years (5.2 years) and most individuals had coinsurance for delivery (94 251 [77.0%]). Price searching increased from 5.9% in the 2011 to 2012 cohort to 13.0% in the 2015 to 2016 cohort. In the 2015 to 2016 cohort, 43.9% of searchers’ first price query was in their first trimester. The adjusted probability of searching was lower for individuals with a high-risk pregnancy due to a previous cesarean delivery (11.5%; 95% CI, 11.0%-12.1%) vs individuals with low-risk pregnancy (13.4%; 95% CI, 12.9%-14.0%). Use increased monotonically with coinsurance, from 9.2% (95% CI, 8.7%-9.8%) among individuals with no coinsurance to 15.0% (95% CI, 14.4%-15.5%) among individuals with 11% or higher coinsurance. After adjusting for covariates, searching was positively associated with out-of-pocket delivery episode spending. Among patients with 11% coinsurance or higher, early and late searchers spent more out of pocket ($59.57 [95% CI, $33.44-$85.96] and $73.33 [95% CI, $32.04-$115.29], respectively), compared with never searchers.

**Conclusions and Relevance:**

The results of this cross-sectional study indicate that the proportion of pregnant individuals who sought price information before childbirth more than doubled within the first 6 years of availability of a price transparency tool. These findings suggest that price information may help individuals anticipate their out-of-pocket childbirth costs.

## Key Points

**Question:**

What are the patterns and characteristics of price transparency tool use among pregnant individuals with commercial insurance?

**Findings:**

In this cross-sectional study of 253 606 pregnant individuals, price transparency tool use increased from 5.9% in the 2011 to 2012 study period to 13.0% in the 2015 to 2016 study period. Higher coinsurance was associated with more price transparency tool use, whereas a previous cesarean delivery was associated with less use.

**Meaning:**

Use of the price transparency tool was associated with higher childbirth spending; these tools may be used for informational and planning purposes by pregnant individuals.

## Teaser

This cross-sectional study examines the association between use of an insurance-owned patient-facing price estimator tool by pregnant individuals and out-of-pocket childbirth spending.

## Introduction

Price transparency continues to be a federal and state policy priority in the United States, as part of efforts to help patients and other purchasers incorporate health care prices into their choice of clinicians and facilities.^1,2^ Since 2010, health care price transparency tools that provide estimates of out-of-pocket and total costs at specific facilities have become increasingly available to patients (eFigure 1 in the Supplement). The most sophisticated of these tools quote episode-level (ie, including all medical services for an episode of care) out-of-pocket prices that account for the patient’s health insurance coverage and deductible spending to date.

Research that was conducted after the introduction of patient-facing price transparency tools in the early 2010s found that few consumers used them.^3,4,5,6^ Studies showed that use of these tools was associated with lower spending on imaging services but not on other services.^4,7,8,9,10,11^ Nonetheless, price estimates for some medical services were queried more frequently than others. These so-called shoppable services tended to be routine, have higher out-of-pocket costs, and be nonemergent (eg, imaging services), allowing patients time to seek price information as they planned for care.

Childbirth, specifically medical care for a vaginal or cesarean delivery of a newborn, is a shoppable service, given the long length of gestation.^3^ It is also the most common reason for hospitalization in the United States.^11,12^ Half of the 3.8 million deliveries each year are covered by private insurance (most others are covered by Medicaid), and 98% of those with private insurance have cost sharing for delivery.^13,14^ In 2015, the mean out-of-pocket spending on maternity services among commercially insured individuals was $4500.^14^

Little is known about how searching for out-of-pocket costs of delivery by pregnant individuals has changed since the introduction of health care price transparency tools. In this cross-sectional study, we used data spanning the 6 years after the introduction of an insurer’s price transparency tool to examine how pregnant individuals used price information on delivery and to add to our understanding of the function of price transparency for shoppable services. Specifically, we sought to measure changes over time in the patterns and characteristics of use of a price transparency tool by pregnant individuals and to identify the association between price transparency tool use, coinsurance, and delivery spending.

## Methods

We followed the Strengthening the Reporting of Observational Studies in Epidemiology (STROBE) reporting guideline. This study was determined to be exempt by the institutional review board at the Harvard T.H. Chan School of Public Health. This institutional review board also waived informed consent because the study was determined to be of minimal risk to participants.

### Context, Design, Data, and Sample

We conducted a descriptive study of 2 cohorts from a national commercial health insurance company that launched a web-based price transparency tool in 2010. The price transparency tool operates with a proprietary claims adjudication logic to provide patients with personalized, episode-level estimates of total and out-of-pocket costs for specific clinicians and facilities for more than 650 medical services, including vaginal and cesarean modes of delivery (eFigure 1 in the Supplement).

We obtained data on all price transparency tool queries, including the services searched and dates of searches, for 2 different periods: January 1, 2011, to December 31, 2012, and January 1, 2015, to December 31, 2016. We linked price queries to administrative enrollment records and medical claims by using unique, deidentified enrollee identification numbers.

We selected all pregnant individuals who had a delivery episode during 2 different 14-month periods: November 1, 2011, to December 31, 2012, or November 1, 2015, to December 31, 2016. The date ranges allowed us to observe all price transparency tool queries during the 10 months before delivery. We defined the date of delivery as the earliest service start date on a claim with a billing code for delivery (eTable 1 in the Supplement). The delivery episode included all paid claims associated with hospitalization on that date and incurred through the last date (ie, service end date or discharge date) associated with the hospitalization claims (eMethods in the Supplement). We identified whether the delivery was vaginal or cesarean (eTable 1 in the Supplement).

We included pregnant individuals aged 19 to 45 years at the start of their delivery episode who were continuously enrolled during the delivery episode and the previous 10 months. We imposed this continuous enrollment requirement to ensure that we observed all price transparency tool queries during pregnancy (eTable 2 in the Supplement).

### Outcomes and Covariates

We classified individuals as searchers if they conducted 1 or more price transparency tool queries for vaginal or cesarean delivery price estimates during the 10 months preceding delivery, and we classified individuals as never searchers if they had no price queries for delivery during that time. Among the searchers, we identified the date of their first price query separately for vaginal delivery and for cesarean delivery. We assigned queries that occurred between 10 calendar months and 189 days before delivery to the first trimester, queries that occurred from 188 days to 98 days before delivery to the second trimester, and queries that occurred from 97 days to 1 day before delivery to the third trimester.

We identified an individual’s age at delivery as the difference in years between their delivery date and date of birth (from the enrollment data). We used the individual’s zip code to identify their hospital referral region (HRR) and whether they lived in an area that was urban, nonurban- and urban-adjacent, or rural.^15,16,17^ We linked the zip codes to data from the American Community Survey^18^ to ascertain the zip code–level educational attainment (high school graduation rate) and median household income in the area (eMethods in the Supplement).

We categorized pregnancy risk as follows: (1) high risk for cesarean delivery attributed to previous cesarean delivery, (2) high risk for cesarean delivery without previous cesarean delivery, (3) presence of other obstetric comorbidities, and (4) low risk. These mutually exclusive risk categories were constructed with *International Classification of Diseases, Ninth Revision* and *International Statistical Classification of Diseases and Related Health Problems, Tenth Revision* codes from established methods.^19,20^ We identified whether an individual had a previous cesarean delivery through diagnosis and procedure codes (eTable 1 in the Supplement). Individuals with a previous cesarean delivery are more likely to have a cesarean delivery with their current pregnancy. We were unable to identify whether any other individuals in the sample had a previous delivery because of data limitations (other diagnosis codes for delivery did not have information on previous vaginal deliveries or parity). We hypothesized that delivery experience and/or increased likelihood of cesarean delivery could affect price transparency tool use; thus, we grouped these individuals in a separate category.

The second risk category included individuals who met the Society for Maternal-Fetal Medicine’s definition of high risk for cesarean delivery but who had not had a previous cesarean delivery.^19^ Individuals who did not meet these criteria but had 1 or more conditions that were captured in the Obstetric Comorbidity Index, including maternal age of 35 years or older, during the 10 months before delivery composed the third risk category group.^20^ The remaining individuals were classified under the fourth risk category of low-risk pregnancy.

We calculated total delivery episode spending as the sum of the allowed amount on each claim during the delivery episode and out-of-pocket spending as the sum of the deductible, coinsurance, and copayment amounts during the delivery episode. We measured coinsurance exposure during delivery as the sum of all coinsurance payments during the delivery episode divided by total delivery episode spending minus any deductible spending.

### Statistical Analysis

We calculated the percentage of the study sample who queried the price transparency tool overall and by delivery mode in 2011 to 2012 and in 2015 to 2016. We compared the differences between the 2 cohorts using a 2-sample χ^2^ test for equality of proportions.

The remaining analyses were conducted on each cohort separately. Results from the 2015 to 2016 cohort are reported in the main text given its relative recency and to provide new evidence on the function of price transparency for this shoppable service. Results from the 2011 to 2012 cohort are provided in eFigure 2, eFigure 3, and eTable 5 in the Supplement.

We examined patterns of price transparency tool use, including whether the delivery mode searched aligned with the delivery outcome, and the timing of searching relative to delivery. We performed multivariable logistic regression to analyze whether rates of searching varied by individual characteristics such as age at delivery, rurality, pregnancy risk status, coinsurance exposure, and the educational attainment quartile and median household income quartile associated with zip codes. The multivariable logistic regression model included fixed effects for month of delivery and HRR and clustered SEs at the HRR level.

We estimated the association between price transparency tool use and delivery spending by running linear regression models with log-transformed total and out-of-pocket delivery episode spending as the dependent variables. The key independent variable was a categorical variable that indicated searcher type: never searcher, early searcher (first searched before the third trimester), or late searcher (first searched during the third trimester). Analysis of total delivery episode spending was stratified by delivery mode. Analysis of out-of-pocket delivery episode spending controlled for delivery mode and was stratified by coinsurance for delivery (ie, 1%-5%, 6%-10%, or ≥11%). Individuals with 0% coinsurance were excluded from the out-of-pocket spending analysis because nearly half of these individuals (46.3%) had no out-of-pocket spending on the delivery episode. Linear regression models controlled for the individual characteristics that were associated with searching in the multivariable logistic regression analysis (age at delivery, rurality, area median household income quartile, and pregnancy risk status). The SEs were clustered at the HRR level. Results of these models are presented as percentage differences in spending and mean dollar differences relative to the reference group (never searchers). We used likelihood ratio tests to compare the estimated coefficients for early and late searchers.

We estimated whether searcher type was associated with delivering at a facility with case-mix and market-adjusted total delivery episode spending above the 50th percentile of all hospitals within the individual’s HRR (a higher-cost facility). Logistic regression was performed for this analysis (eMethods in the Supplement).

All hypothesis tests were 2-sided, with a significance level of α = .05. Data analyses were conducted from March 2020 to February 2021, using R, version 3.6.1 (R Foundation for Statistical Computing).

## Results

The sample comprised 253 606 pregnant individuals, of whom 131 224 (51.7%) were in the 2011 to 2012 cohort and 122 382 (48.3%) were in the 2015 to 2016 cohort. The demographic characteristics were similar across the 2 cohorts. In the 2015 to 2016 cohort, the mean (SD) age was 31 years (5.2 years) and most individuals had coinsurance for delivery (94 251 [77.0%]). Most individuals in both cohorts lived in areas that were urban, were in the top 2 quartiles of educational attainment, and were in the top 2 quartiles of median household income (Table 1). Approximately 1 in 5 individuals had had a previous cesarean delivery. Most individuals (64.1% in the 2015-2016 cohort) faced higher than 5% coinsurance exposure for their delivery episode, and a similar proportion (65.2% in the 2015-2016 cohort) incurred more than $1000 of out-of-pocket costs for the delivery episode.

**Table 1.** Characteristics of the 2011-2012 and 2015-2016 Cohorts

Characteristic	No. (%)	
	2011-2012 Cohort	2015-2016 Cohort
Delivery episodes, No.	131 224	122 382
Age at delivery, y		
19-24	15 677 (12.0)	12 833 (10.5)
25-29	32 454 (24.7)	28 207 (23.1)
30-34	48 642 (37.1)	47 201 (38.6)
35-39	27 282 (20.8)	27 797 (22.7)
40-45	7169 (5.5)	6344 (5.2)
Rurality		
Urban	110 905 (84.5)	99 962 (81.7)
Nonurban and urban-adjacent	16 691 (12.7)	17 849 (14.6)
Rural	3628 (2.8)	4571 (3.7)
Area educational attainment quartile^a^		
Highest	46 191 (35.2)	38 421 (31.4)
High	37 353 (28.5)	37 726 (30.8)
Low	27 156 (20.7)	25 513 (20.9)
Lowest	19 556 (14.9)	19 632 (16.0)
Missing data	968 (0.7)	1090 (0.9)
Area median household income quartile^b^		
Highest	75 106 (57.2)	71 260 (58.2)
High	26 943 (20.5)	25 537 (20.9)
Low	18 480 (14.1)	17 161 (14.0)
Lowest	9751 (7.4)	7227 (5.9)
Missing data	944 (0.7)	1197 (1.0)
Pregnancy risk status		
High risk for cesarean delivery with previous cesarean delivery	23 248 (17.7)	22 389 (18.3)
High risk for cesarean delivery without previous cesarean delivery^c^	42 971 (32.8)	21 255 (17.4)
Presence of other obstetric comorbidities^c^	25 481 (19.4)	43 799 (35.8)
Low risk	39 524 (30.1)	34 939 (28.6)
Out-of-pocket spending for delivery episode, $		
0	13 028 (9.9)	13 169 (10.8)
1-1000	45 755 (34.9)	29 422 (24.0)
>1000	72 441 (55.2)	79 791 (65.2)
Coinsurance for delivery episode, %		
0	38 173 (29.1)	28 131 (23.0)
1-5	12 467 (9.5)	15 828 (12.9)
6-10	36 325 (27.7)	30 566 (25.0)
≥11	44 258 (33.7)	47 857 (39.1)

^a^ American Community Survey data were used to create national quartiles of the percentage of people 25 years or older without a high school diploma in a zip code. These quartiles were linked to the individuals’ zip codes. See eMethods in the Supplement for details.

^b^ American Community Survey data were used to create national quartiles of median household income at the zip code level. These quartiles were linked to the individuals’ zip codes. See eMethods in the Supplement for details.

^c^ The difference in the proportions of the cohorts across risk categories can be attributed to the differences between the *International Classification of Diseases, Ninth Revision* (2011-2012 cohort) and *International Statistical Classification of Diseases and Related Health Problems, Tenth Revision* (2015-2016 cohort) codes available to classify these conditions.

**Figure 1.**
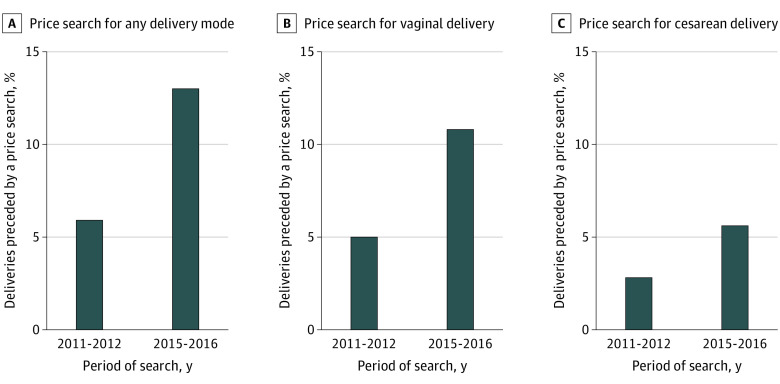
Rates of Searching for Price Estimates for Any, Vaginal, or Cesarean Delivery Among Pregnant Individuals, 2011-2012 and 2015-2016 In the 2011 to 2012 cohort (n = 131 224), 7781 pregnant individuals (5.9%) searched price estimates for any delivery mode, 6511 (5.0%) for vaginal delivery, and 3648 (2.8%) for cesarean delivery. In the 2015 to 2016 cohort, (n = 122 382), 15 936 pregnant individuals (13.0%) searched price estimates for any delivery mode, 13 187 (10.8%) for vaginal delivery, and 6883 (5.6%) for cesarean delivery. The difference between the 2011 to 2012 and 2015 to 2016 search rates was statistically significant within each category (*P* < .001).

Use of the price transparency tool before delivery increased from the 2011 to 2012 cohort to the 2015 to 2016 cohort (5.9% vs 13.0%; *P* < .001) (Figure 1). Rates of searching for price estimates also increased from the 2011 to 2012 cohort to the 2015 to 2016 cohort for vaginal delivery (5.0% vs 10.8%; *P* < .001) and for cesarean delivery (2.8% vs 5.6%; *P* < .001).

Most of the results from the 2015 to 2016 cohort were qualitatively consistent with the results from the 2011 to 2012 cohort. We present results from the 2015 to 2016 cohort; the 2011 to 2012 cohort results are shown in eFigure 2, eFigure 3, and eTable 5 in the Supplement.

### Search Timing and Delivery Mode

Searchers queried price estimates for vaginal delivery only (56.8% of searchers), cesarean delivery only (17.3%), or both delivery modes (25.9%). Searchers and never searchers had similar delivery outcomes (eg, vaginal delivery: 65.8% and 67.9%, respectively) (eTable 3 in the Supplement). Those who searched for price information on vaginal delivery only were much more likely to have a vaginal delivery than those who searched for cesarean delivery only (69.2% vs 33.0%).

**Figure 2.**
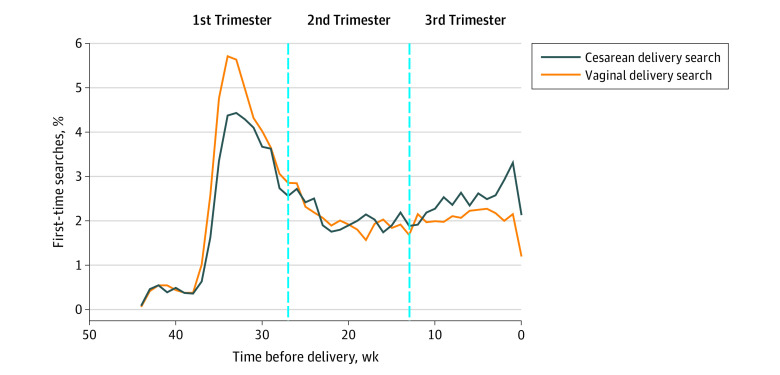
Weeks Before Delivery at Time of First Price Transparency Tool Search by Delivery Mode, 2015-2016 Trimesters were based on a 40-week gestational period, and the date of delivery was defined as the start of hospital admission. The first trimester was defined as 10 months to 189 days before delivery; second trimester, as 188 to 98 days before delivery; and third trimester, as 97 to 1 day before delivery. The x-axis indicates the number of weeks before delivery at which the pregnant individual first used the price transparency tool for each delivery mode.

Nearly half (43.9%) of searchers first queried the price transparency tool in the first trimester of pregnancy (Figure 2 and eFigure 2 in the Supplement). First-time queries for cesarean delivery prices increased in the 10 weeks before delivery. Among individuals who searched for cesarean delivery price estimates for the first time in the third trimester, only 19.7% had searched for vaginal delivery price estimates before their first cesarean delivery price search.

### Association Between Individual Characteristics and Searching

**Figure 3.**
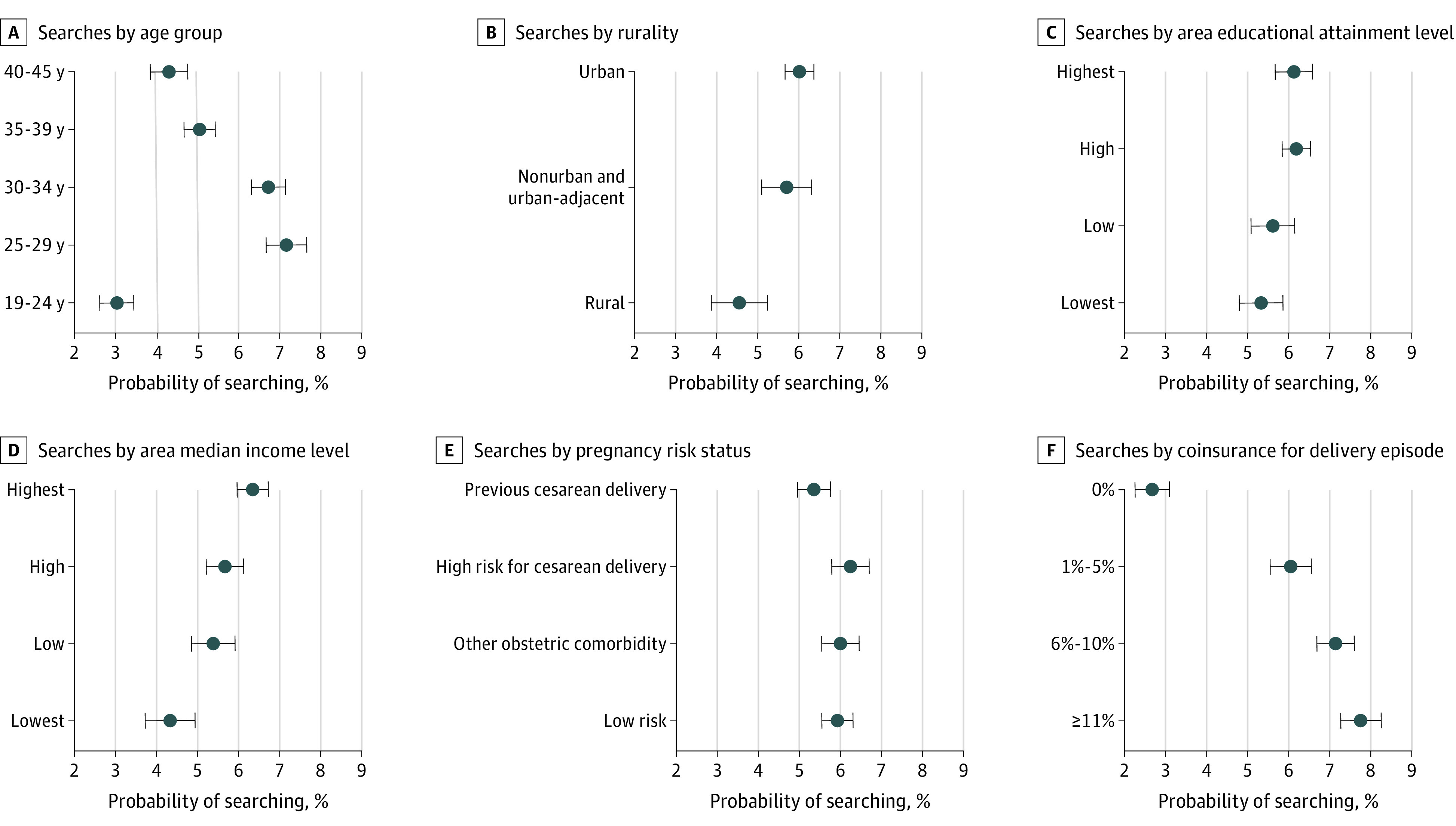
Adjusted Probability of Price Transparency Tool Use During Pregnancy by Individual Characteristics, 2015-2016 Estimated probabilities and 95% CIs were calculated with a logistic regression model, regressing an indicator for searching on the characteristics displayed and month and hospital referral region (HRR) fixed effects. Standard errors were clustered at the HRR level.

Individuals in the middle of the age distribution (aged 25-34 years) were more likely to search for delivery prices than younger (aged 19-24 years) or older (aged 35-45 years) individuals (Figure 3 and eFigure 3 in the Supplement; eTable 4 in the Supplement shows the unadjusted results). Price transparency tool use increased with urbanicity and median household income of the individuals’ area of residence. Use was lower among individuals with high-risk pregnancies due to previous cesarean delivery compared with individuals in all other risk groups (eg, 11.5%; 95% CI, 11.0%-12.1% vs 13.4%; 95% CI, 12.9%-14.0% among low-risk individuals). The adjusted probability of searching increased monotonically with coinsurance exposure for the delivery episode, from 9.2% (95% CI, 8.7%-9.8%) among individuals with no coinsurance to 15.0% (95% CI, 14.4%-15.5%) among individuals with 11% or higher coinsurance.

### Association Between Searching and Delivery Spending

Searching was positively associated with out-of-pocket delivery episode spending (Table 2 and eTable 5 in the Supplement) and with total delivery episode spending (eTable 6 in the Supplement). Among individuals with the highest percentage of coinsurance, early and late searchers spent more out of pocket ($59.57 [95% CI, $33.44-$85.96] and $73.33 [95% CI, $32.04-$115.29], respectively) compared with never searchers. This pattern and the magnitude of the spending differences were similar for other coinsurance levels (Table 2). No statistically significant difference in out-of-pocket or total delivery episode spending was found between early and late searchers.

**Table 2.** Out-of-Pocket Spending by Coinsurance Percentage and Timing of Search, 2015-2016^a^

Spending	Percentage of coinsurance and timing of search								
	1%-5% (n = 15 687)			6%-10% (n = 30 361)			≥11% (n = 47 458)		
	Never	Early	Late	Never	Early	Late	Never	Early	Late
Mean out-of-pocket spending, $	623.66	681.96	766.46	1446.45	1529.12	1573.09	2486.97	2560.36	2592.16
% Change in out-of-pocket spending (95% CI)	0 [Reference]	6.8 (1.1-12.8)	14.5 (6.6-22.9)	0 [Reference]	4.3 (2.4-6.1)	5.8 (3.4-8.3)	0 [Reference]	2.4 (1.3-3.5)	2.9 (1.3-4.6)
Mean difference (95% CI), $	0 [Reference]	42.35 (6.64- 80.08)	90.38 (41.43-142.94)	0 [Reference]	61.74 (35.39-88.57)	83.75 (48.93-119.38)	0 [Reference]	59.57 (33.44- 85.96)	73.33 (32.04-115.29)
*P* value^b^		<.001	<.001		<.001	<.001		<.001	<.001

^a^ Model results are from linear regression models as described in the Methods section. Delivery episodes with missing values for area median income were excluded from the analysis. Likelihood ratio tests failed to reject the null hypothesis that the coefficients on early and late searchers were equal. The percentage change in out-of-pocket spending was calculated by exponentiating the coefficient from the model and converting it into a percentage; the dollar change multiplied that percentage by the mean out-of-pocket spending in the never searcher group.

^b^ *P* values were relative to the reference category (never searcher).

Late searchers were more likely than never searchers to deliver at a higher-cost facility (55.2% [95% CI, 53.7%-56.7%] vs 52.8% [95% CI, 52.5%-53.2%]) (eFigure 4 in the Supplement). There was no difference between early searchers and never searchers in the probability of delivering at a higher-cost facility.

## Discussion

Among a national sample of commercially insured pregnant individuals, use of a price transparency tool for delivery doubled in the first 6 years the tool was available, from 5.9% in 2011 to 2012 to 13.0% in 2015 to 2016. This 6-year period corresponded with increasing out-of-pocket costs for maternity services,^14^ and this finding is consistent with patient surveys reporting that awareness of price transparency tools improved between 2010 and 2017.^6,21,22^

Use of the price transparency tool appeared responsive to clinical information about delivery mode. Much higher rates of vaginal delivery were found among individuals who searched only for vaginal delivery price estimates compared with those who searched only for cesarean delivery price estimates. We observed an increase in first-time searches for cesarean delivery in the third trimester; this time is when individuals may receive clinical information about a likely cesarean delivery, such as fetal presentation.

Individuals with higher levels of coinsurance were more likely to use the price transparency tool. These individuals generally have a stronger incentive to obtain price estimates because they pay a higher proportion of total delivery costs than other individuals do. Searching may also be affected by the number of in-network childbirth facility options that pregnant individuals have. We observed lower rates of searching in rural areas and areas with lower median household income, which are more likely to have fewer facilities offering hospital-based obstetric services.^23^

We observed that individuals who had a previous cesarean delivery were less likely to conduct a price search. These individuals may have an existing relationship with a clinician or may be committed to delivering at a particular facility that they perceive to be suited to caring for more complex cases. Most price transparency tools display limited, if any, information on quality of care that could assist patients in evaluating cost and quality together to choose a higher-value, rather than just lower-priced, clinician or facility. Studies of clinician selection among pregnant individuals have reported that most of these individuals select their clinician, obstetrician, or midwife and then deliver at whichever hospital or birthing center the clinician has admitting privileges.^24^

We found a positive association between using the price transparency tool and delivery spending. Although this association cannot be interpreted as a causal relationship between searching and spending, it raises important hypotheses about the unobserved characteristics of searchers that are also associated with higher delivery spending. The results of this study suggest that this association is unlikely to be attributed to searchers selecting higher-cost facilities compared with nonsearchers. Searching late in the pregnancy, but not searching before the third trimester, was associated with delivering at a high-cost facility. By the time they conducted a search in the third trimester, late searchers were likely to have already selected a facility for childbirth. Instead, those who had planned to give birth at a relatively high-cost facility may have been aware of the expected costs and may have used the price transparency tool for financial planning. We cannot rule out the possibility that late searchers preferred higher-cost facilities because they equated higher cost with higher quality. However, surveys have found that most people in the United States understand that health care costs and quality are not necessarily correlated.^25,26^

Early searchers, who were not more likely than never searchers to deliver at a high-cost facility, may be different from never searchers in some way that is associated with delivery cost within a facility. Early searchers may have had higher costs because of having a higher risk pregnancy in ways that we could not measure in our data. Perhaps they preferred more health care services within the delivery episode. Previous research has generally shown that price transparency tools are not associated with selection of lower-priced facilities^7,8,9,10,11^; more studies are needed to trace whether this finding would continue to be true over time.

Use of price transparency tools that is not associated with selection of lower-priced clinicians or facilities does not necessarily mean the tools are of no value. Individuals may use the tool for informational or financial planning purposes. Health care price information can help patients anticipate their out-of-pocket expenditures.^27^ Individuals may also perform a price search to verify that a facility is within their network to protect against high out-of-pocket costs or surprise bills.^22,28^ Future research can examine whether price transparency has helped patients prepare for their out-of-pocket costs and improved downstream financial outcomes.

### Limitations

This study has several limitations. First, we examined only 1 price transparency tool, and the results may not be generalizable to tools that may be designed differently or marketed and offered by other insurers, employers, government, or third parties. Second, the data we obtained did not capture whether individuals in the sample used other price transparency tools. Third, the results may not be generalizable to services other than delivery, although focusing on this shoppable service provides insights into price transparency in the context of high out-of-pocket costs. Fourth, the results may not be generalizable to pregnant individuals who are uninsured, are covered by Medicaid, use the price transparency tool to select a nonhospital setting for delivery, or experience insurance churn during pregnancy.^29^ Fifth, there are likely unobserved differences between searchers and never searchers that may be associated with delivery spending; therefore, these findings cannot be interpreted as causal.

## Conclusions

Searching for childbirth price information increased over the first 6 years after the launch of a price transparency tool but remains uncommon. Interest in price estimates was highest early in pregnancy, although early searchers were not more likely than never searchers to deliver at a lower-cost facility. The findings of this cross-sectional study suggest that price transparency tools can be used for informational or financial planning purposes in addition to clinician and facility selection. Promoting the use of price transparency tools for these purposes may increase their use and could help individuals plan for high out-of-pocket costs.

## Notes

Supplement. **eFigure 1.** Mockup Screen Shots of the Price Transparency Tool **eTable 1.** Diagnosis and Procedure Codes Used to Identify and Classify Delivery Episodes **eMethods.** Additional Detail on Measures and Analyses **eTable 2.** Study Sample Selection and Attrition Table **eTable 3.** Searching by Final Delivery Mode and Delivery Mode Searched for, 2015-2016 and 2011-2012 **eFigure 2.** Weeks Before Delivery at Time of First Price Transparency Tool Search, by Delivery Mode, 2015-2016 **eFigure 3.** Adjusted Probability of Price Transparency Tool Use During Pregnancy by Individual Characteristics, 2011-2012 **eTable 4.** Unadjusted Demographic Differences Between Searchers and Never-Searchers for 2011-2012 and 2015-2016 Cohorts **eTable 5.** Out-of-Pocket Spending by Coinsurance Percentage and Timing of Search, 2011-2012 **eTable 6.** Total Spending by Coinsurance Percentage and Final Delivery Mode, 2011-2012 and 2015-2016 **eFigure 4.** Predicted Probability of Delivering at High-Cost Facility by Searcher Type, 2011-2012 and 2015-2016
