# Supplementary material for: Patterns of Use of a Price Transparency Tool for Childbirth Among Pregnant Individuals With Commercial Insurance
Source: JAMA Netw Open. 2021 Aug 18;4(8):e2121410. doi: 10.1001/jamanetworkopen.2021.21410 (PMC8374613; doi:10.1001/jamanetworkopen.2021.21410)
Supplement: Supplement. — eFigure 1. Mockup Screen Shots of the Price Transparency Tool eTable 1. Diagnosis and Procedure Codes Used to Identify and Classify Delivery Episodes eMethods. Additional Detail on Measures and Analyses eTable 2. Study Sample Selection and Attrition Table eTable 3. Searching by Final Delivery Mode and Delivery Mode Searched for, 2015-2016 and 2011-2012 eFigure 2. Weeks Before Delivery at Time of First Price Transparency Tool Search, by Delivery Mode, 2015-2016 eFigure 3. Adjusted Probability of Price Transparency Tool Use During Pregnancy by Individual Characteristics, 2011-2012 eTable 4. Unadjusted Demographic Differences Between Searchers and Never-Searchers for 2011-2012 and 2015-2016 Cohorts eTable 5. Out-of-Pocket Spending by Coinsurance Percentage and Timing of Search, 2011-2012 eTable 6. Total Spending by Coinsurance Percentage and Final Delivery Mode, 2011-2012 and 2015-2016 eFigure 4. Predicted Probability of Delivering at High-Cost Facility by Searcher Type, 2011-2012 and 2015-2016 [file jamanetwopen-e2121410-s001.pdf]

Supplementary Online Content

Gourevitch RA, Chien AT, Bambury EA, et al. Patterns of use of a price transparency tool for childbirth among pregnant individuals with commercial insurance. *JAMA Netw Open*. 2021;4(8):e2121410. doi:10.1001/jamanetworkopen.2021.21410

eFigure 1. Mockup Screen Shots of the Price Transparency Tool

eTable 1. Diagnosis and Procedure Codes Used to Identify and Classify Delivery Episodes

eMethods. Additional Detail on Measures and Analyses

eTable 2. Study Sample Selection and Attrition Table

eTable 3. Searching by Final Delivery Mode and Delivery Mode Searched for, 2015-2016 and 2011-2012

eFigure 2. Weeks Before Delivery at Time of First Price Transparency Tool Search, by Delivery Mode, 2015-2016

eFigure 3. Adjusted Probability of Price Transparency Tool Use During Pregnancy by Individual Characteristics, 2011-2012

eTable 4. Unadjusted Demographic Differences Between Searchers and Never-Searchers for 2011-2012 and 2015-2016 Cohorts

eTable 5. Out-of-Pocket Spending by Coinsurance Percentage and Timing of Search, 2011-2012

eTable 6. Total Spending by Coinsurance Percentage and Final Delivery Mode, 2011-2012 and 2015-2016

eFigure 4. Predicted Probability of Delivering at High-Cost Facility by Searcher Type, 2011-2012 and 2015-2016

This supplementary material has been provided by the authors to give readers additional information about their work.

© 2021 Gourevitch RA et al. *JAMA Network Open.*

eFigure 1. Mockup Screen Shots of the Price Transparency Tool

Step 1: Enter the procedure you are searching for

Search for a procedure to estimate costs

Vaginal delivery

Step 2: Enter your ZIP code

Enter your ZIP code

12345

Name	Address & Phone	Distance	Your Estimated Cost
Hospital 1	123 Smith Street<br>123-456-7890	10.26 miles	$2,616.05<br>View Details
Hospital 2	456 Main Street<br>234-567-8901	5.75 miles	$2,817.44<br>View Details
Hospital 3	789 Broadway<br>345-678-9012	3.19 miles	$4,019.52<br>View Details
…			
Hospital 10	258 Clover Ave<br>456-789-0123	21.24 miles	$3,330.24<br>View Details

© 2021 Gourevitch RA et al. *JAMA Network Open.*

Step 4: Click “View Details” on a hospital you are interested in.

Hospital 1

Your plan pays: $10,719.02

Your total estimated payment: **$2,616.05**

Your cost breakdown	
Amount paid toward meeting your deductible	$300.00
Copayment	$0.00
Coinsurance payment	$2,316.04
Services your plan doesn’t cover	$0.00

Note: the information displayed is be based on your benefit design, the amount of spending you’ve had to date in your plan year, and your plan’s negotiated rates with the hospital.

© 2021 Gourevitch RA et al. *JAMA Network Open.*

eTable 1. Diagnosis and Procedure Codes Used to Identify and Classify Delivery Episodes

**Code**	**Description**	**Vaginal delivery**	**Cesarean delivery**	**Previous Cesarean delivery**
**ICD-9 Diagnosis Code**				
650	Normal delivery	X		
649.81	Spontaneous labor with plan for C/S delivery, with or without mention of antepartum condition		X	
649.82	Spontaneous labor with plan for C/S delivery, with mention of postpartum complication		X	
669.71	Cesarean delivery, without mention of indication, delivered, with or without mention of antepartum condition		X	
654.2	Previous cesarean delivery, unspecified as to episode of care or not applicable			X
**ICD-9 Procedure Code**				
72	Forceps, vacuum, and breech delivery	X		
73.22; 73.59	Other manually assisted deliveries	X		
73.6	Episiotomy	X		
74.	Cesarean section and removal of fetus		X	
**ICD-10 Diagnosis Code**				
O80	Encounter for full-term uncomplicated delivery	X		
O66.41	Failed attempted vaginal birth after previous cesarean delivery		X	X
O86.13	Vaginitis following delivery	X		
O75.82	Spontaneous labor with delivery by planned cesarean section		X	
O82	Encounter for cesarean delivery without indication		X	
O90.0	Disruption of cesarean delivery wound		X	
P03.4	Newborn affected by cesarean delivery		X	
O34.21	Maternal care for scar from previous cesarean delivery			X
**ICD-10 Procedure Code**				
10E0XZZ	Delivery of products of conception, external approach	X		
10D07Z3	Extraction of products of conception, low forceps, via natural or artificial opening	X		
10D07Z4	Extraction of products of conception, mid forceps, via natural or artificial opening	X		
10D07Z5	Extraction of products of conception, high forceps, via natural or artificial opening	X		
10D07Z6	Extraction of products of conception, vacuum, via natural or artificial opening	X		
10D00Z0	Extraction of products of conception, high, open approach		X	
10D00Z1	Extraction of products of conception, low, open approach		X	
10D00Z2	Extraction of products of conception, extraperitoneal, open approach		X	
**CPT Code**				
59400	Routine obstetric care including antepartum care, vaginal delivery (with or without episiotomy, and/or forceps) and postpartum care	X		

© 2021 Gourevitch RA et al. *JAMA Network Open.*

59409	Vaginal delivery only (with or without episiotomy and/or forceps)	X		
59410	Vaginal delivery only (with or without episiotomy and/or forceps); including postpartum care	X		
59610	Routine obstetric care including antepartum care, vaginal delivery (with or without episiotomy, and/or forceps) and postpartum care, after previous cesarean delivery	X		
59612	Vaginal delivery only, after previous cesarean delivery (with or without episiotomy and/or forceps)	X		
59614	Vaginal delivery only, after previous cesarean delivery (with or without episiotomy and/or forceps); including postpartum care	X		
59510	Routine obstetric care including antepartum care, cesarean delivery, and postpartum care		X	
59514	Cesarean delivery only		X	
59515	Cesarean delivery only; including postpartum care		X	
59618	Routine obstetric care including antepartum care, cesarean delivery, and postpartum care, following attempted vaginal delivery after previous cesarean delivery		X	
59620	Cesarean delivery only, following attempted vaginal delivery after previous cesarean delivery		X	
59622	Cesarean delivery only, following attempted vaginal delivery after previous cesarean delivery; including postpartum care		X	
**MS-DRG Code**				
767	Vaginal delivery w sterilization &/or D&C	X		
768	Vaginal delivery w OR procedure except sterilization &/or D&C	X		
774	Vaginal delivery w complicating diagnoses	X		
775	Vaginal delivery w/o complicating diagnoses	X		
765	Cesarean delivery w cc/mcc		X	
766	Cesarean delivery w/o cc/mcc		X	

© 2021 Gourevitch RA et al. *JAMA Network Open.*

eMethods. Additional Detail on Measures and Analyses

<u>Additional detail on defining the delivery episode</u>.

Most claim lines for inpatient care had a *date service started* and an *admission date*, as well as a *date service stopped* and a *discharge date*. We defined the <u>delivery date</u> as the earliest *date service started* on a claim with a billing code for delivery (eTable 1). We refer to this as <u>first delivery claim line</u>. If that claim line also had an *admission date* and a *discharge date*, we defined the start of the episode as their *admission date* and the end of the episode as the last *discharge date* associated with the corresponding *admission date*.

If the <u>first delivery claim line</u> did not include an *admission date* and/or a *discharge date,* we used the following logic to assign a start and end date to the delivery episode:

If *admission date* attached to <u>first delivery claim line</u> AND no *discharge date* attached to *admission date*:

- Start of episode = *admission date*
- End of episode = last *date service stopped* attached to *admission date* within 30 days of *date service started*

If no *admission date* attached to <u>first delivery claim line</u>, but admitted 1 day prior to delivery:

- Start of episode = *admission date* 1 day prior to delivery
- End of episode = last *discharge date* equal to or later than <u>delivery date</u> attached to *admission date* 1 day prior to delivery

If no *admission date* attached to <u>first delivery claim line</u>, but admitted 1 day after delivery:

- Start of episode = <u>delivery date</u>
- End of episode = last *discharge date* attached to *admission date* 1 day after delivery

If no *admission date* attached to <u>first delivery claim line</u> and not admitted within ± 1 days of delivery

- Start of episode = <u>delivery date</u>
- End of episode = last *discharge date* attached to any claims with a code for vaginal or cesarean delivery (eTable 1)

If no *admission date* attached to <u>first delivery claim line</u>, not admitted within ± 1 days of delivery, and had no *discharge date* attached to any claims with code for vaginal or cesarean delivery

- Start of episode = <u>delivery date</u>
- End of episode = 2 days after <u>delivery date</u> if vaginal delivery or 4 days after <u>delivery date</u> if cesarean delivery

<u>Additional detail on ZIP-code level covariates</u>.

We used American Community Survey (ACS) data from the U.S. Census Bureau to obtain 5-year nationally representative estimates of demographic information at the ZIP code level. We sorted all ZIP codes according to their median household income from the ACS into quartiles (1st quartile = lowest income, 4th quartile = highest income). We repeated this process for each ZIP code by percent of adults ages 25 and older without a high school degree (1st quartile = lowest educational attainment, 4th quartile = highest educational attainment). We then linked patient ZIP codes to these quartiles. We used 2011-2015 ACS data for the 2011-2012 cohort and 2014-2018 ACS data for the 2015-2016 cohort.

<u>Additional detail on the high-cost facility analysis</u>.

We analyzed whether searching early or late in pregnancy was associated with selecting a higher-cost facility within a patient's HRR. This analysis was restricted to members whose ZIP code matched to area median income information from the American Community Survey and who had a delivery claim with a de-identified hospital identifier (N=124,227 (95% of sample) for 2011-2012 and N=111,135 (91% of sample) for 2015-2016). We only included patients who delivered at a facility with a least 10 deliveries and who lived in an HRR with at least four hospitals in the sample study period. The sample size for the analysis was N=118,780 deliveries (91% of the full

© 2021 Gourevitch RA et al. *JAMA Network Open.*

sample) across 1,500 hospitals for 2011-2012 and N=105,199 deliveries (86% of the full sample) across 1,396 hospitals for 2015-2016. We calculated each facility's case-mix and market-adjusted average total delivery spending using a linear model regressing log-transformed total delivery spending, Winsorized at the 5th and 95th percentiles, on facility and HRR fixed effects (controlling for pregnancy risk status, delivery mode, age group, rurality, quartile of area median income, and month of delivery); standard errors were clustered at the HRR-level.

We determined whether each facility's median adjusted predicted total delivery spending was above or below the median adjusted predicted total delivery spending among members in each HRR. We created an indicator variable at the patient-level for whether they went to a facility that was below or above the median adjusted total spending in their HRR. Finally, we ran a logistic regression model with the dependent variable indicating whether the member went to a high- or low-cost facility and the independent variable of whether they searched early in pregnancy, late in pregnancy, or never in pregnancy. Results of the model are reported as predicted probabilities.

© 2021 Gourevitch RA et al. *JAMA Network Open.*

eTable 2. Study Sample Selection and Attrition Table

	2011-2012 COHORT		2015-2016 COHORT	
**Inclusion/exclusion criteria**	**N Remaining**	**Percent dropped from prior step**	**N Remaining**	**Percent dropped from prior step**
All members who gave birth during study period (see Appendix 1 for billing codes)[a]	189,130		205,975	
Drop claims where status of claim not = “Paid” or member Zip code = “Unknown”	187,025	1.11%	204,085	0.92%
Drop reversed claim lines	187,013	0.01%	204,027	0.03%
Members with a delivery claim remaining in study period[b]	180,586	3.44%	186,977	8.36%
Drop members with claims attached to babies (delivery year – birth year = 0)	180,472	0.06%	175,772	5.99%
Continuously enrolled for 10 months prior to delivery	140,847	21.96%	128,421	26.94%
Had valid ZIP code, RUCA value and HRR	140,726	0.09%	128,359	0.05%
Had one age record, and was 19-45 years old at delivery	138,475	1.60%	127,026	1.04%
Had billing codes for either vaginal or cesarean delivery (not both)	131,446	5.08%	122,796	3.33%
Had total delivery episode spending > $0	131,263	0.14%	122,433	0.30%
Belong to an HRR with greater than 10 members	131,224	0.03%	122,382	0.04%

Notes:
a. If a patient had more than one delivery within either time period, we excluded the second delivery from analysis to avoid autocorrelation between episodes.
b. We believe the difference in percent dropped across the two cohorts at this step is due to differences in coding for live birth deliveries between ICD-9 and ICD-10 coding time frames. We see more live birth delivery codes applied to neonate members for 2015-2016 members than 2011-2012 members.

eTable 3. Searching by Final Delivery Mode and Delivery Mode Searched for, 2015-2016 and 2011-2012

**2015-2016 COHORT**			
		By Final Delivery Mode, N (%)	
	Total, N	Vaginal	Cesarean
Total sample	122,382	82,716 (67.6)	39,666 (32.4)
Total never-searchers	106,446	72,231 (67.9)	34,215 (32.1)
Total searchers	15,936	10,485 (65.8)	5,451 (34.2)
Delivery mode searched for, N (%)			
Vaginal only	9,053 (56.8)	7,254 (69.2)	1,799 (33.0)
Cesarean only	2,749 (17.3)	558 (5.3)	2,191 (40.2)
Both vaginal and cesarean	4,134 (25.9)	2,673 (25.5)	1,461 (26.8)
**2011-2012 COHORT**			
		By Final Delivery Mode, N (%)	
	Total, N	Vaginal	Cesarean
Total sample	131,224	87,274 (66.5)	43,950 (33.5)
Total never-searchers	123,443	82,211 (66.6)	41,232 (33.4)
Total searchers	7,781	5,063 (65.1)	2,718 (34.9)
Delivery mode searched for, N (%)			
Vaginal only	4,133 (53.1)	3,342 (66.0)	781 (28.7)
Cesarean only	1,270 (16.3)	155 (3.1)	1,115 (41.0)
Both vaginal and cesarean	2,378 (30.6)	1,566 (30.9)	812 (29.9)

© 2021 Gourevitch RA et al. *JAMA Network Open.*

eFigure 2. Weeks Before Delivery at Time of First Price Transparency Tool Search, by Delivery Mode, 2015-2016

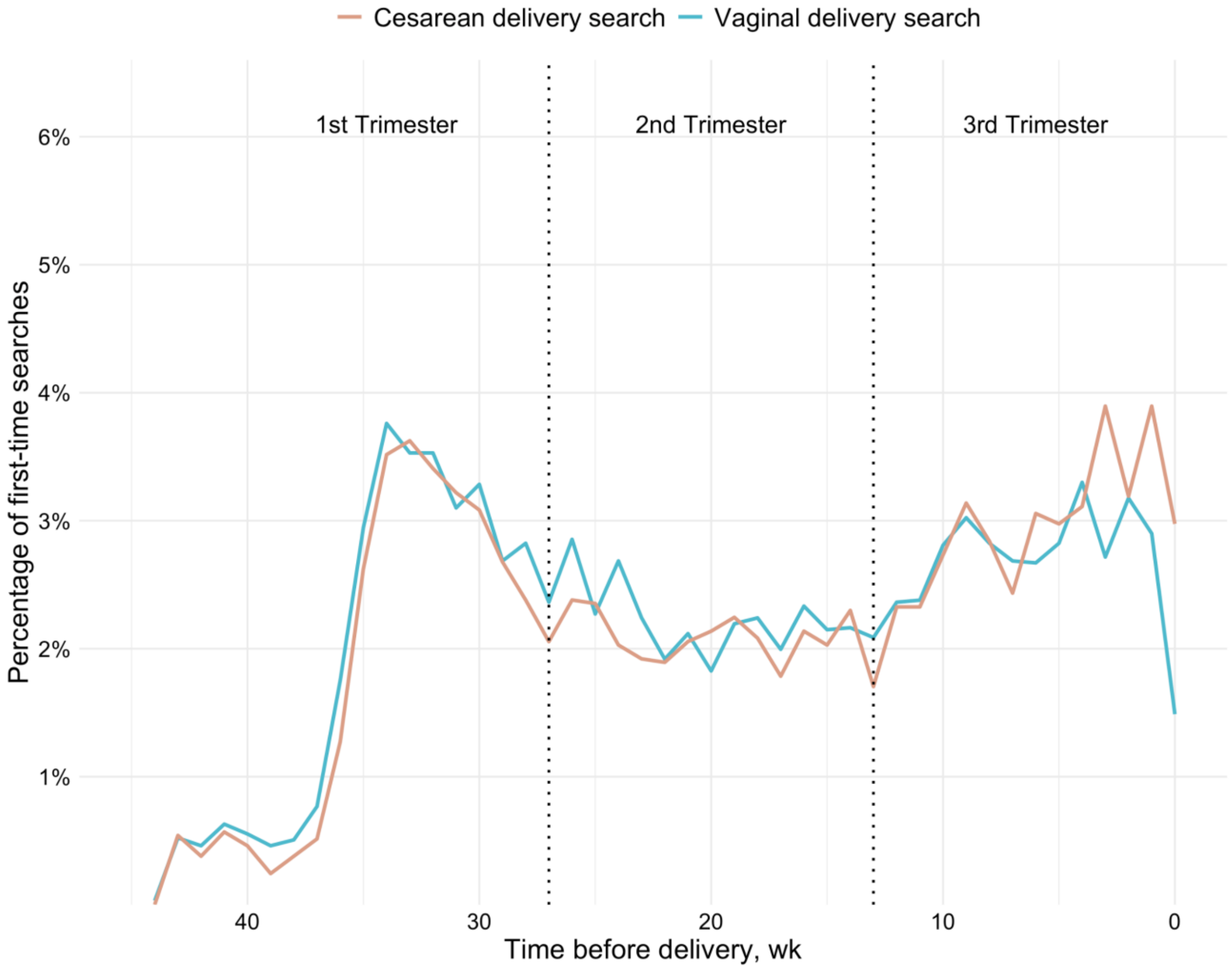

Note: Trimesters were based on a 40-week gestational period, and the date of delivery was defined as the start of hospital admission. The first trimester was defined as 10 months to 189 days before delivery; second trimester as 188 to 98 days before delivery; and third trimester as 97 to 1 day before delivery. The x-axis indicates the number of weeks before delivery at which the patient first used the price transparency tool for each delivery mode.

© 2021 Gourevitch RA et al. *JAMA Network Open.*

eFigure 3. Adjusted Probability of Price Transparency Tool Use During Pregnancy by Individual Characteristics, 2011-2012

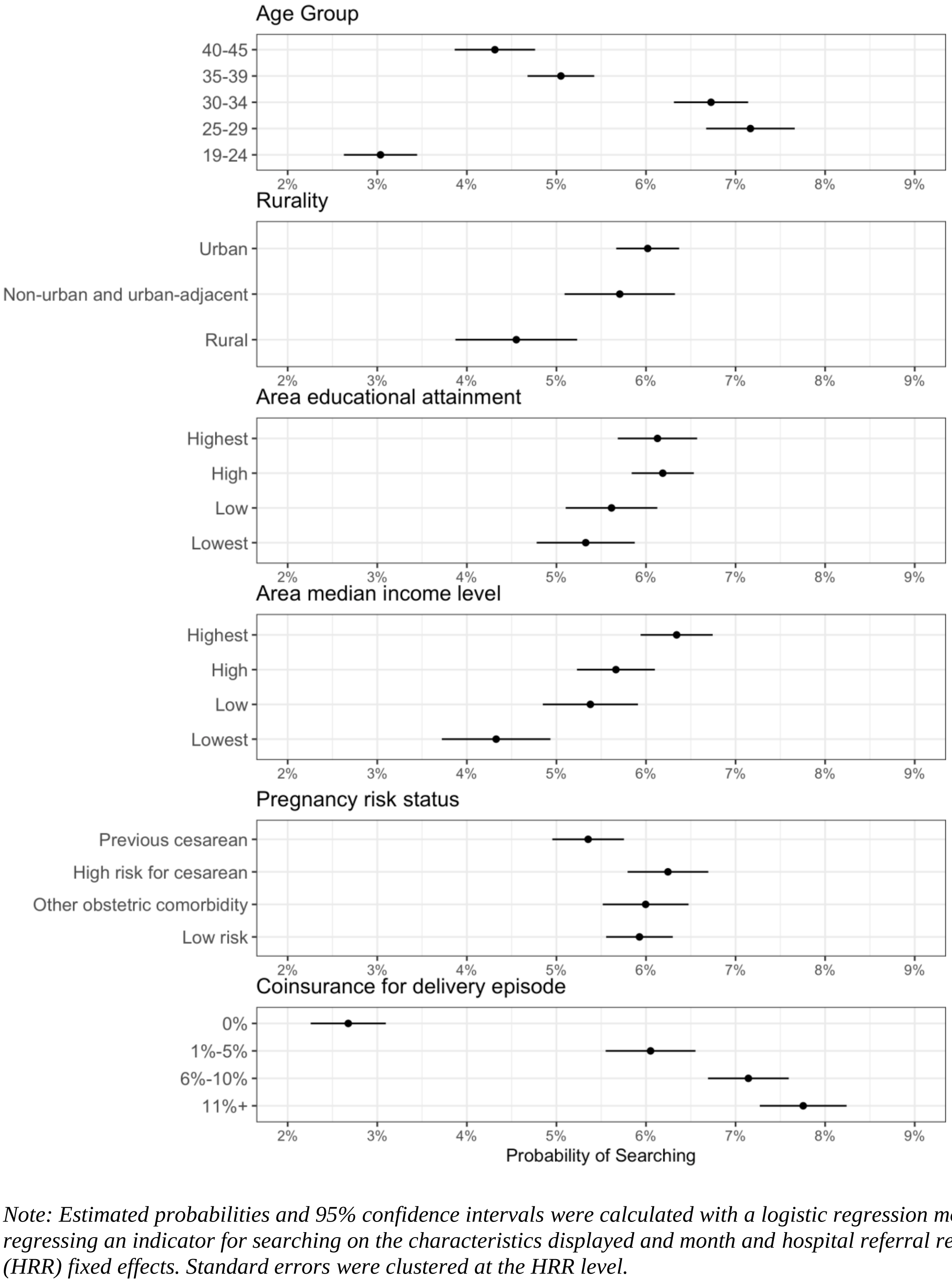

Note: Estimated probabilities and 95% confidence intervals were calculated with a logistic regression model, regressing an indicator for searching on the characteristics displayed and month and hospital referral region (HRR) fixed effects. Standard errors were clustered at the HRR level.

© 2021 Gourevitch RA et al. *JAMA Network Open.*

eTable 4. Unadjusted Demographic Differences Between Searchers and Never-Searchers for 2011-2012 and 2015-2016 Cohorts

	**2011-2012 COHORT**				**2015-2016 COHORT**			
	Total (%)	Searchers (%)	Never-Searchers (%)	p-value	Total (%)	Searchers (%)	Never-Searchers (%)	p-value
Delivery episodes (N (%))	131,224	7,781 (5.9%)	123,443 (94.1%)		122,382	15,936 (13.0%)	106,446 (87.0%)	
Age (years)								
19-24	12.0	5.7	12.3	<0.001	10.5	4.6	11.4	<0.001
25-29	24.7	30.0	24.4		23.1	26.8	22.5	
30-34	37.1	42.6	36.7		38.6	43.8	37.8	
35-39	20.8	17.8	21.0		22.7	21.1	23.0	
40-45	5.5	3.9	5.6		5.2	3.8	5.4	
Rurality								
Urban	84.5	85.8	84.4	<0.001	81.7	84.5	81.3	<0.001
Non-urban and urban-adjacent	12.7	12.3	12.7		14.6	13.0	14.8	
Rural	2.8	1.9	2.8		3.7	2.5	3.9	
Area educational attainment								
Highest	35.2	39.2	35.0	<0.001	31.4	33.8	31.0	<0.001
High	28.5	30.6	28.3		30.8	32.7	30.6	
Low	20.7	18.4	20.8		20.9	19.4	21.1	
Lowest	14.9	11.3	15.1		16.0	13.8	16.4	
Missing	0.7	0.5	0.8		0.9	0.4	1.0	
Area median income								
Highest	57.2	63.1	56.9	<0.001	58.2	63.1	57.5	<0.001
High	20.5	19.6	20.6		20.9	20.5	20.9	
Low	14.1	12.1	14.2		14.0	11.7	14.4	
Lowest	7.4	4.7	7.6		5.9	4.2	6.2	
Missing	0.7	0.5	0.7		1.0	0.5	1.1	
Pregnancy risk status								
Previous cesarean delivery	17.7	16.0	17.8	<0.001	18.3	16.2	18.6	<0.001
High risk for cesarean delivery	32.8	34.4	32.6		17.4	17.7	17.3	
Other obstetric comorbidities	19.4	18.1	19.5		35.8	35.4	35.9	
Low risk	30.1	31.5	30.0		28.6	30.7	28.2	
Out-of-pocket spending on delivery								
$0	9.9	5.8	10.2	<0.001	10.8	6.4	11.4	<0.001
$1-$1,000	34.9	24.5	35.5		24.0	19.9	24.7	
> $1,000	55.2	69.7	54.3		65.2	73.7	63.9	
Coinsurance for delivery episode								
0%	29.1	13.1	30.1	<0.001	23.0	16.3	24.0	<0.001
1%-5%	9.5	9.9	9.5		12.9	12.5	13.0	
6%-10%	27.7	33.9	27.3		25.0	27.2	24.6	
11%+	33.7	43.1	33.1		39.1	44.0	38.4	

Note: p-value is chi2 p-value between searchers and never-searcher groups.

© 2021 Gourevitch RA et al. *JAMA Network Open.*

eTable 5. Out-of-Pocket Spending by Coinsurance Percentage and Timing of Search, 2011-2012

	Percentage Coinsurance and Timing of Search								
	**1-5% (n = 12,386)**			**6-10% (n = 36,094)**			**≥11% (n = 43,912)**		
	Never	*Early*	*Late*	*Never*	*Early*	*Late*	*Never*	*Early*	*Late*
Mean out-of-pocket spending, $	503.72	547.21	593.8	1201.81	1277.31	1267.41	2039.02	2087.51	2102.71
% Change in out-of-pocket spending (95% CI)	0 [Reference]	-0.2 (-0.2, 16.9)	6.5 (6.5, 26.4)	0 [Reference]	5.7 (3.7, 7.7)	4.9 (2.5, 7.3)	0 [Reference]	2.1 (0.1, 4.2)	2.5 (0.2, 4.8)
Mean difference (95% CI), $	0 [Reference]	40.38 (-0.78, 84.91)	80.62 (32.6, 132.94)	0 [Reference]	68.04 (44.24, 92.3)	58.5 (30.5, 87.14)	0 [Reference]	42.81 (1.67, 84.77)	50.64 (3.53, 98.84)
P-Value		0.055	0.001		<0.001	<0.001		0.041	0.035

Note: Model results are from linear regression models as described in the Methods section. Delivery episodes with missing values for area median income 6 were excluded from the analysis. Likelihood ratio tests failed to reject the null hypothesis that the coefficients on early and late searchers were equal. The percentage change in out-of-pocket spending was calculated by exponentiating the coefficient from the model and converting it into a percentage; the dollar change multiplied that percentage by the mean out-of-pocket spending in the never searcher group.

© 2021 Gourevitch RA et al. *JAMA Network Open.*

eTable 6. Total Spending by Coinsurance Percentage and Final Delivery Mode, 2011-2012 and 2015-2016

**2015-2016 COHORT**						
	**Vaginal Delivery (N=81,886)**			**Cesarean Delivery (N=39,299)**		
	Never	*Early*	*Late*	*Never*	*Early*	*Late*
Mean total spending, $	10587.71	11083.85	11258.19	15219.14	15614.51	15946.62
% Change in total spending (95% CI)	0 [Reference]	3.5 (2.1, 4.9)	4.4 (3, 6)	0 [Reference]	1.1 (-0.3, 2.5)	1.9 (0.3, 3.6)
Mean difference (95% CI), $	0 [Reference]	371.12 (225.82, 518.36)	470.85 (313.07, 630.92)	0 [Reference]	167.29 (-40.64, 378.08)	293.81 (43.26, 548.47)
P-Value		<0.001	<0.001		0.115	0.021
**2011-2012 COHORT**						
	**Vaginal Delivery (N=86,630)**			**Cesarean Delivery (N=43,650)**		
	Never	*Early*	*Late*	*Never*	*Early*	*Late*
Mean total spending, $	9185.05	9272.64	9506.35	13497.45	13557.29	13575.92
% Change in total spending (95% CI)	0 [Reference]	0 (-1.8, 1.9)	2 (0.2, 3.9)	0 [Reference]	-0.5 (-2.6, 1.8)	-0.9 (-3.4, 1.6)
Mean difference (95% CI), $	0 [Reference]	2.31 (-164.94, 172.66)	186.21 (19.54, 355.89)	0 [Reference]	-60.96 (-353.76, 238.36)	-123.57 (-457.08, 218.47)
P-Value		0.979	0.028		0.687	0.475

Note: results are from a linear model regressing log-transformed and Winsorized total delivery spending on type of searcher, controlling for pregnancy risk status, age group, rurality, quartile of area median income, and with fixed effects for month of delivery and HRR. Separate models were run for vaginal and cesarean delivery episodes. Delivery episodes with missing values for area median income were excluded from the analysis. Standard errors are clustered at the HRR level. P-values reported relative to reference category (never searched the transparency tool). Likelihood ratio tests failed to reject the null hypothesis that the coefficients on early and late searchers were equal. The percent change in total spending is the coefficient from the model exponentiated and converted into a percent; the dollar change multiplies that percent by the average out-of-pocket spending in the never-searcher group. Early searchers first searched before their third trimester; late searchers first searched during their third trimester.

© 2021 Gourevitch RA et al. *JAMA Network Open.*

eFigure 4. Predicted Probability of Delivering at High-Cost Facility by Searcher Type, 2011-2012 and 2015-2016

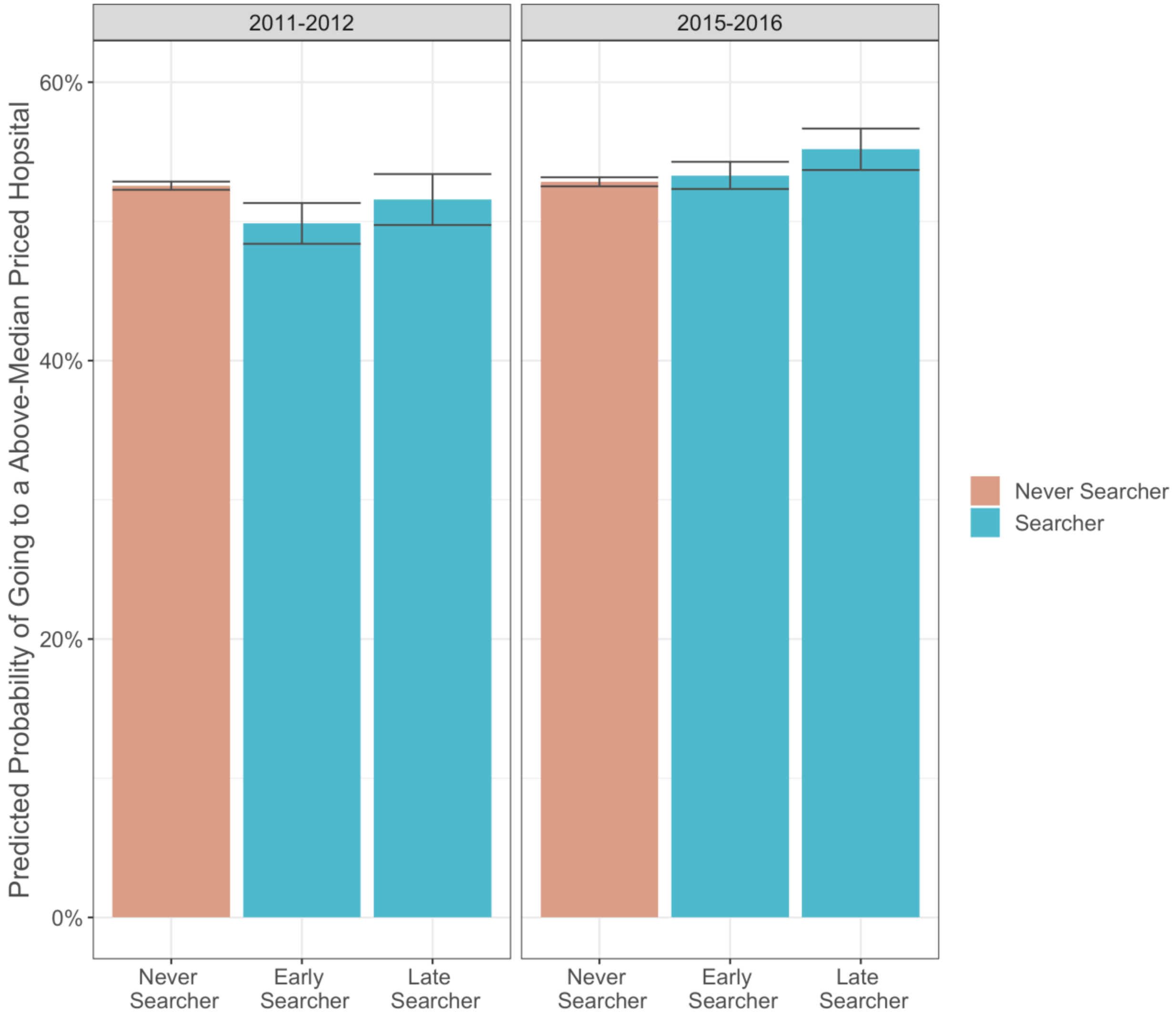

Notes: Results are shown as predicted probabilities from logistic regression of an indicator for delivering at a hospital above the median for total delivery spending in an HRR on searcher type. See eMethods for additional details. Early searchers first searched before their third trimester; late searchers first searched during their third trimester.

© 2021 Gourevitch RA et al. *JAMA Network Open.*
